# Finite element analysis of the treatment of a minimally invasive approach combined with a novel anatomical locking plate for scapular body fractures

**DOI:** 10.1186/s13018-024-04905-7

**Published:** 2024-07-17

**Authors:** Zhanpeng Guo, Yue Guo, Yansong Wang, Yunlong Bi, Yu Deng, Yang Cao, Mina Huang

**Affiliations:** 1 Department of Orthopedics, The First Affiliated Hospital of Jinzhou Medical University, Jinzhou, China; 2 School of Nursing, Jinzhou Medical University, Jinzhou, China

**Keywords:** Scapular body fractures, Finite element analysis, Anatomical locking plate, Reconstructive plate, Internal fixation

## Abstract

**Background:**

The minimally invasive approach for the treatment of displaced scapular neck or body fractures has the advantages of less trauma and minimal muscle dissection. In clinical practice, the minimally invasive approach combined with an anatomical locking plate has been used to treat scapular body fractures. In addition, we have made minor modifications to the minimally invasive approach. However, the biomechanical study about the approach combined with an anatomical locking plate in treating scapular body fractures was limited.

**Methods:**

Finite element analysis (FEA) was used to conduct the biomechanical comparison between the anatomical locking plate (AP model) and reconstructive plate (RP model) in the treatment of scapular body fractures through the modified minimally invasive approach. A healthy male volunteer with no history of scapula or systemic diseases was recruited. High-resolution computed tomography images of his right scapula were obtained. Two scapula models were constructed and analyzed by the software of Mimics 21.0, Geomagic Wrap 2021, SolidWorks 2021, and ANSYS Workbench 2022, respectively.

**Results:**

Through static structural analysis, in terms of equivalent von Mises stress, equivalent elastic strain, and total deformation, the AP model exhibited superior safety characteristics, enhanced flexibility, and anticipated stability compared with the RP model. This was evidenced by lower maximum stress, lower maximum strain and displacement.

**Conclusion:**

The minimally invasive approach combined with an anatomical locking plate for scapular body fractures had better biomechanical stability. The study provided a biomechanical basis to guide the clinical treatment of scapular body fractures.

## Introduction

Scapular fractures are bone injuries that occur in the scapula, a triangular flat bone that connects the collarbone to the upper arm bone [1]. Scapular fractures are uncommon and typically occur in conjunction with severe trauma [2]. It has been found to occur at a rate of 0.7% of all fractures [3]. Anatomical classification allows for the categorization of scapular fractures into four distinct types: fracture of the acromion, fracture of the scapular body fractures, fracture of the scapular neck, and fracture of the inferior angle of the scapula [4].

Scapular fractures have been regarded as high-energy fractures [5]. The main classified methods for scapular fractures include the AO and Miller classifications [6–8]. The Miller classification currently stands as the prevailing method for categorizing scapular fractures [8]. Most scapular fractures can achieve good function with conservative treatment [9, 10]. However, for some scapular fractures with large displacements or multiple injuries, early surgical treatment can facilitate functional recovery. Rollo et al. [11] demonstrated that surgical treatment can lead to improved functional outcomes in the short term for extraarticular scapular fractures. Studies also have found that patients with scapular fractures who undergo surgery can obtain positive outcomes [9, 12]. A related study pointed out that surgical intervention was effective in achieving favourable functional outcomes and minimizing complications for severe fractures of the scapular body and glenoid neck [12]. The studies also suggested that surgical interventions for unstable scapular fractures could enhance the clinical outcome and the function of the shoulder joint [13, 14].

Currently, the surgical treatment of scapular fractures is generally fixed with reconstruction plates, locking compression plates, distal radius T-plates or screws [2, 8, 15]. Studies showed that the implementation of open reduction and internal fixation for scapular fractures that have become displaced could result in an effective union rate and favourable functional outcomes [16]. The surgical approach for scapular fractures included the modified Judet approach [17], minimally invasive approach [18], reverse Judet approach [19], and mirror Judet approach [20]. Scapular body fractures were usually treated by the posterior inverted 7 approach of the Judet approach, which could be fixed by plate fixation or reconstruction of structural plate [17, 21]. The minimally invasive approach for scapular fractures involving the body, neck, and posterior glenoid has demonstrated its less invasive surgical technique with minimal muscular dissection [18].

Finite element analysis (FEA) is an engineering method that utilizes mathematical simulations to determine the response of a structure or material when exposed to external forces under load [22]. In recent years, FEA has been a widely used method to compare the biomechanical stability of different implants for the treatment of fractures [23, 24]. He et al. [25] investigated a novel dualplate fixation method for proximal humeral fractures without medial support from the finite element viewpoint.

Studies have also explored the mechanical stability of three techniques used in the fixation of transverse and oblique metaphyseal-diaphyseal junction fractures of the distal humerus in children [26]. Zhang et al. [27] also through the finite element analysis indicated that dual small plate fixation may provide a viable option for fixing midshaft clavicle fractures.

Scapula has its unique anatomical structure. The scapula relative to the clavicle can happen on the internal rotation, upper spin, and backward lean [28, 29]. Scapular upward rotation should be contributed to both humeral elevation and axial rotation [29]. In general, the scapula experiences intricate three-dimensional movements that cannot be adequately characterized by rotations around a single anatomical axis [30]. According to the anatomical characteristics of the scapula and its surrounding muscles, we designed a new anatomical locking plate that is more practical. In addition, we made a minor modification to the placement of the steel plates, which was reflected in the placement of the cephalic side of the inner plate on the upper ridge of the scapular spine instead of its lower edge. By improving the internal fixation technique and integrating it with a minimally invasive approach, we have successfully implemented this method in clinical treatment. Although we have confirmed it in clinical practice, further biomechanical validation is essential for comprehensive scientific substantiation.

Given this, in the current study, FEA was used to study and compare the biomechanical stability between anatomical locking plate (AP model) and reconstructive plate (RP model) for treating scapular body fractures by using the minimally invasive approach, aiming to establish a theoretical foundation for future clinical interventions.

## Materials and methods

### Construction of the scapula model

A healthy male volunteer, aged 45 and without any previous scapular injury or systemic diseases, was recruited for the study. The written informed consent was obtained.

**Fig. 1:**
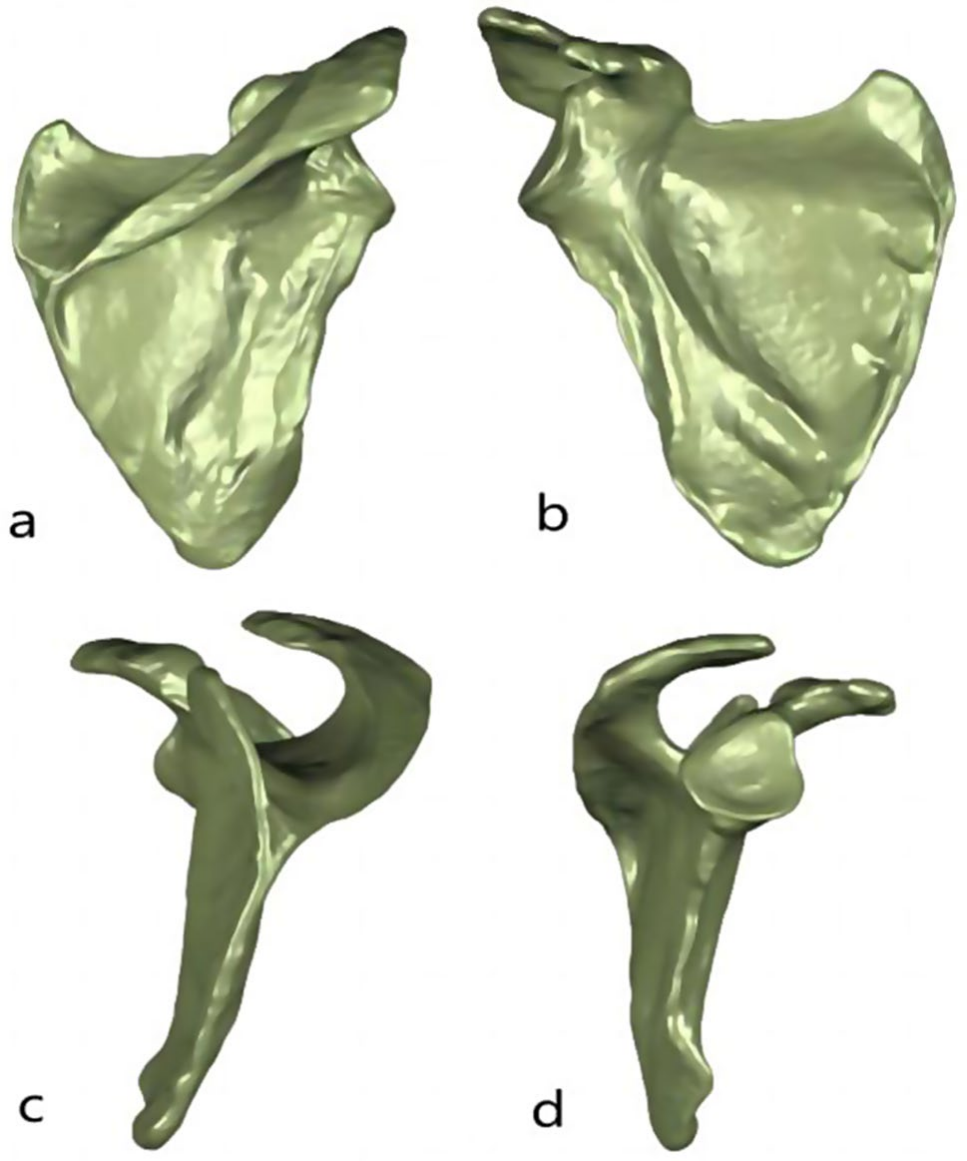
Solid model of the scapula. **a**, Posterior view of the right scapula; **b**, Anterior view of the right scapula; **c** and **d**, Side view of the right scapula

64-slice CT scanner (Sensation64, Siemens, Germany) was utilized to obtain high-resolution computed tomography (CT) images of the right scapula of the volunteer. The images obtained by scanning were stored as DICOM format and then filed into the Mimics 21 software (Materialise, Leuven, Belgium). A preliminary scapula model can be obtained through techniques such as threshold determination and image segmentation. Then the model was saved as STL format. Finally, the model was imported into Geomagic Wrap 2021 software (Geomagic, USA). The solid model of the scapula was initially obtained by applying mesh doctor diagnostics, removal of spikes, accurate surface, etc. The model was saved as STP format (Fig. 1).

### Model construction of scapular body fractures

**Fig. 2:**
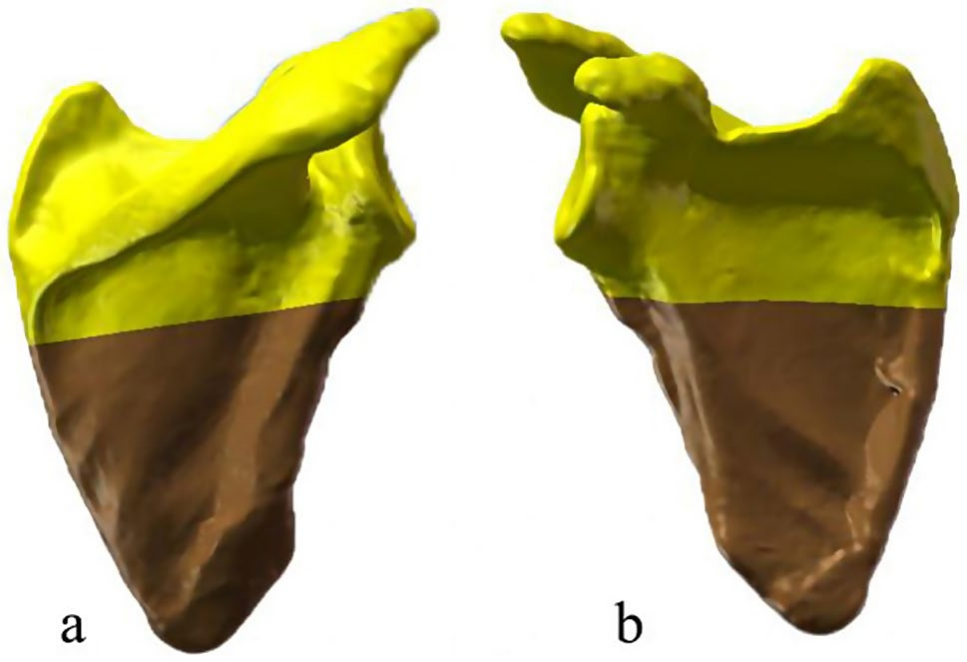
The solid model of the fractured scapula. **a**, Posterior view of the right scapula; **b**, Anterior view of the right scapula

The solid model of the scapula was imported into Solidworks 2021 software (Dassault, France), where it underwent a cutting process along the designated fracture line to generate the fractured solid model (Fig. 2).

### Constructing a model for internal fixation of scapular body fractures

**Fig. 3:**
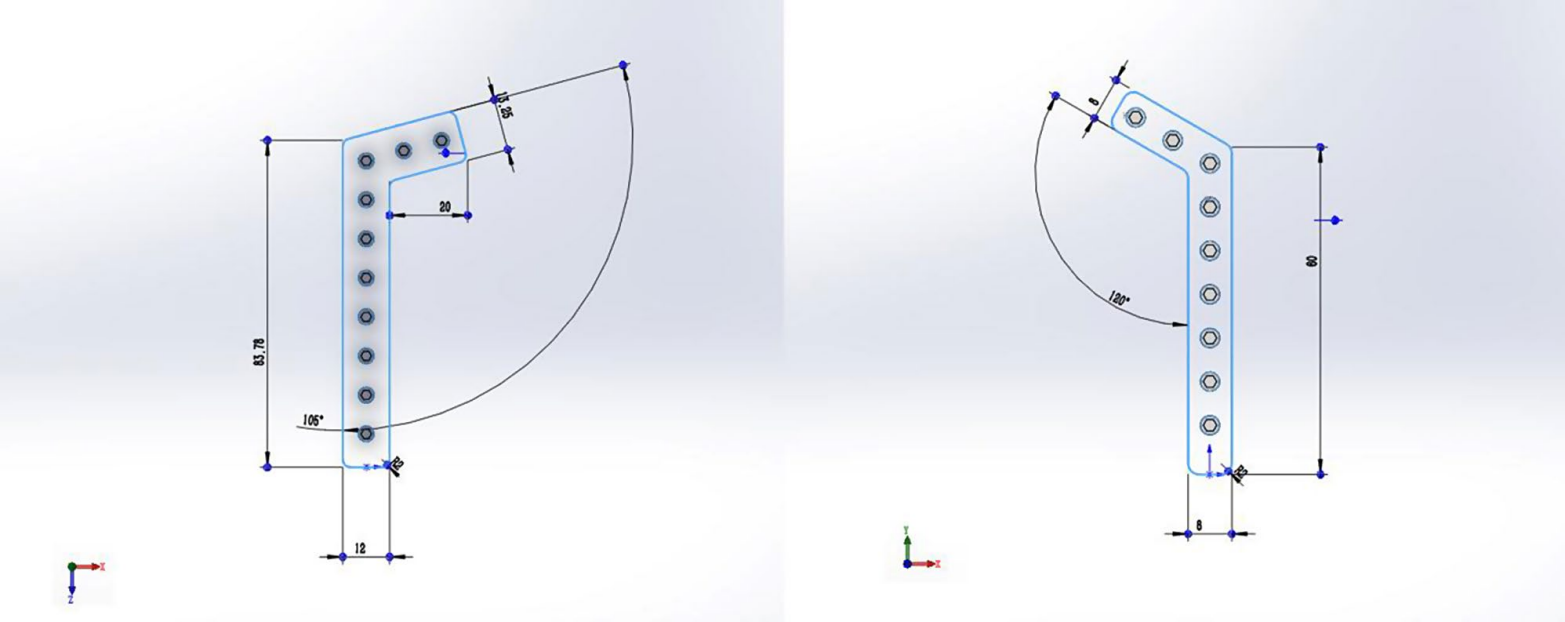
The steel plates of the AP model

**Fig. 4:**
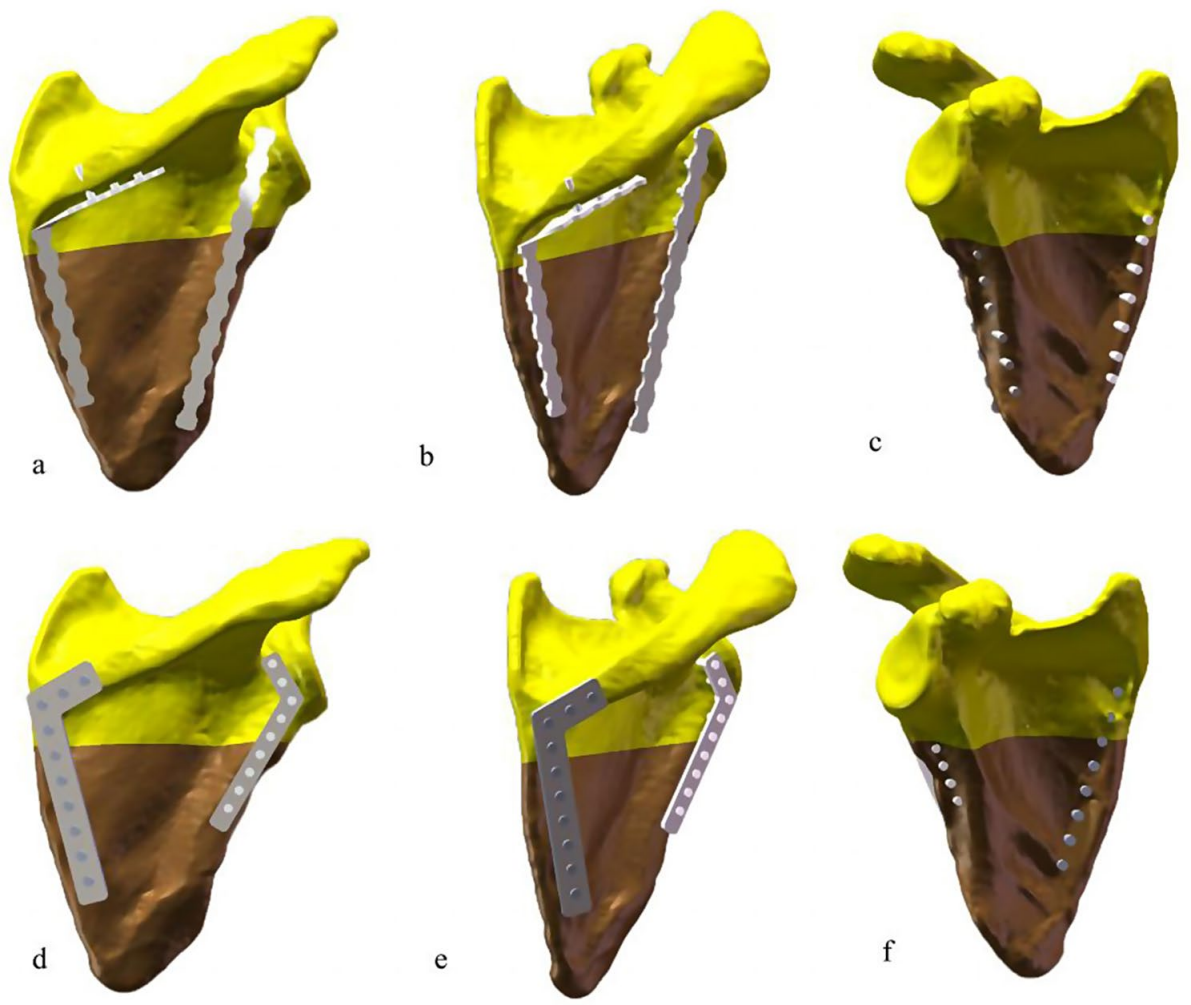
The internal fixation model of the scapular body fractures. RP model (**a**, **b**, **c**); AP model (**d**, **e**, **f**)

Using SolidWorks 2021 software, the internal fixation models (RP model and AP model) were drawn which matched the fracture model, with the scapular body as the reference plane. The steel plates of the AP model were shown in Fig. 3. As shown in Fig. 3, the inner plate has an angle of 105 degrees and a thickness of 2 mm, and the outer plate has an angle of 120 degrees and a thickness of 2 mm. The diameter of the screw was 3.5 mm, and the screw and rod were replaced by a smooth cylinder, omitting the threads. According to the basic norms of surgical operation and the principles of internal fixation, the obtained fracture model and internal fixator were assembled on the assembly interface to obtain the internal fixation model of the scapular body fractures (Fig. 4).

### Volume mesh generation and material properties

**Fig. 5:**
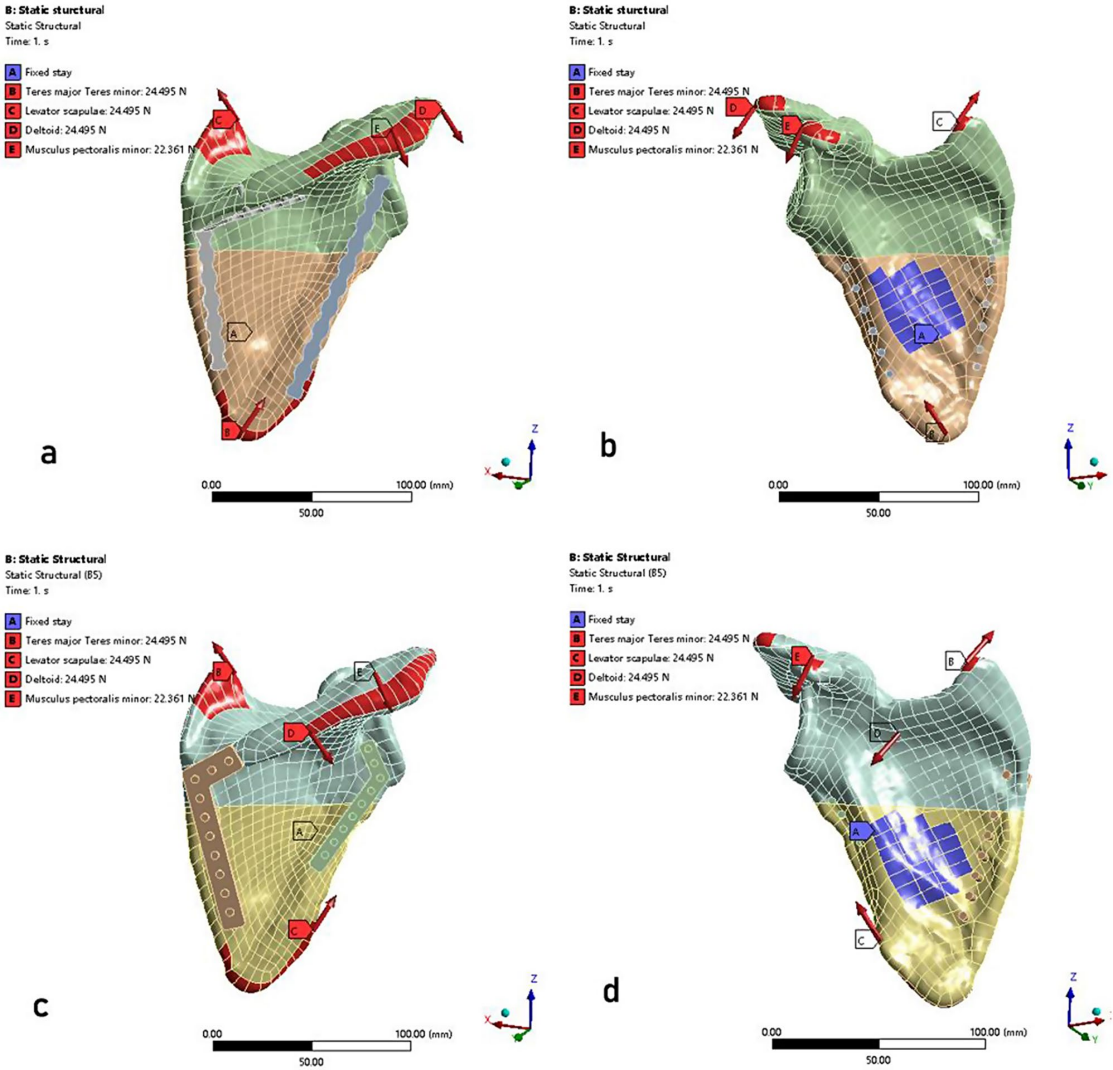
Loading and boundary conditions: A, fixed support, B, teres major and teres minor, C, levator scapulae, D, deltoid, E, musculus pectoralis minor. RP model (**a**, **b**); AP model (**c**, **d**)

The ANSYS Workbench 2022 (Swanson, Houston, USA) was utilized to import the models [31]. The mesh type was set to a tetrahedral mesh with a mesh size of 1.5 mm (Fig. 5). And the number of nodes and elements of the two models were shown in Table 1. Then, the mesh quality was assessed and optimized, ensuring that the convergence analysis of the FEA models achieved a level below 5% [23]. The scapula, screw, and plate were modelled as isotropic, mean-continuous, and linear elastic materials. The material properties which were shown in Table 2 were assigned according to the study by Shang et al. [32].

**Table 1:** Number of nodes and elements of the two models

	RP model	AP model
Number of nodes	288,560	289,517
Number of elements	152,571	153,556

**Table 2:** Material properties of the various components

Materials	Young’s modulus (MPa)	Poisson’s ratio
Cortical bone	17,000	0.30
Cancellous bone	1600	0.30
Steel plate	114,000	0.30
Screw	114,000	0.30

### Boundary conditions and loading force settings

The contact relationships between the fracture sections (cortical bone and cortical bone, cancellous bone and cancellous bone) were modelled as frictional, with a friction coefficient of 0.2 [33]. The friction coefficient between the screw and cortical bone was 0.8, while with cancellous bone was 0.3. The contact relationships involving the locking plate and the screw, as well as those between the cortical bone and the cancellous bone of the scapula, were defined as binding. Muscular forces were applied and the depiction of each site was manifested in Fig. 5. As shown in Fig. 5, the load on the scapula during extension was simulated by teres major and teres minor, levator scapulae, deltoid, and musculus pectoralis minor. Meanwhile, fixed support was implemented at the attachment point of the subscapularis muscles.

### Evaluation criteria

The stress cloud diagram was used to observe and analyze the distribution of von Mises stress and maximum von Mises stress values in each group of models for the scapula and internal fixation. Meanwhile, the displacement cloud diagram was employed to record and analyze model displacements. These parameters were utilized to illustrate the mechanical factors that impact the stability of internal fracture fixation [24].

## Results

**Fig. 6:**
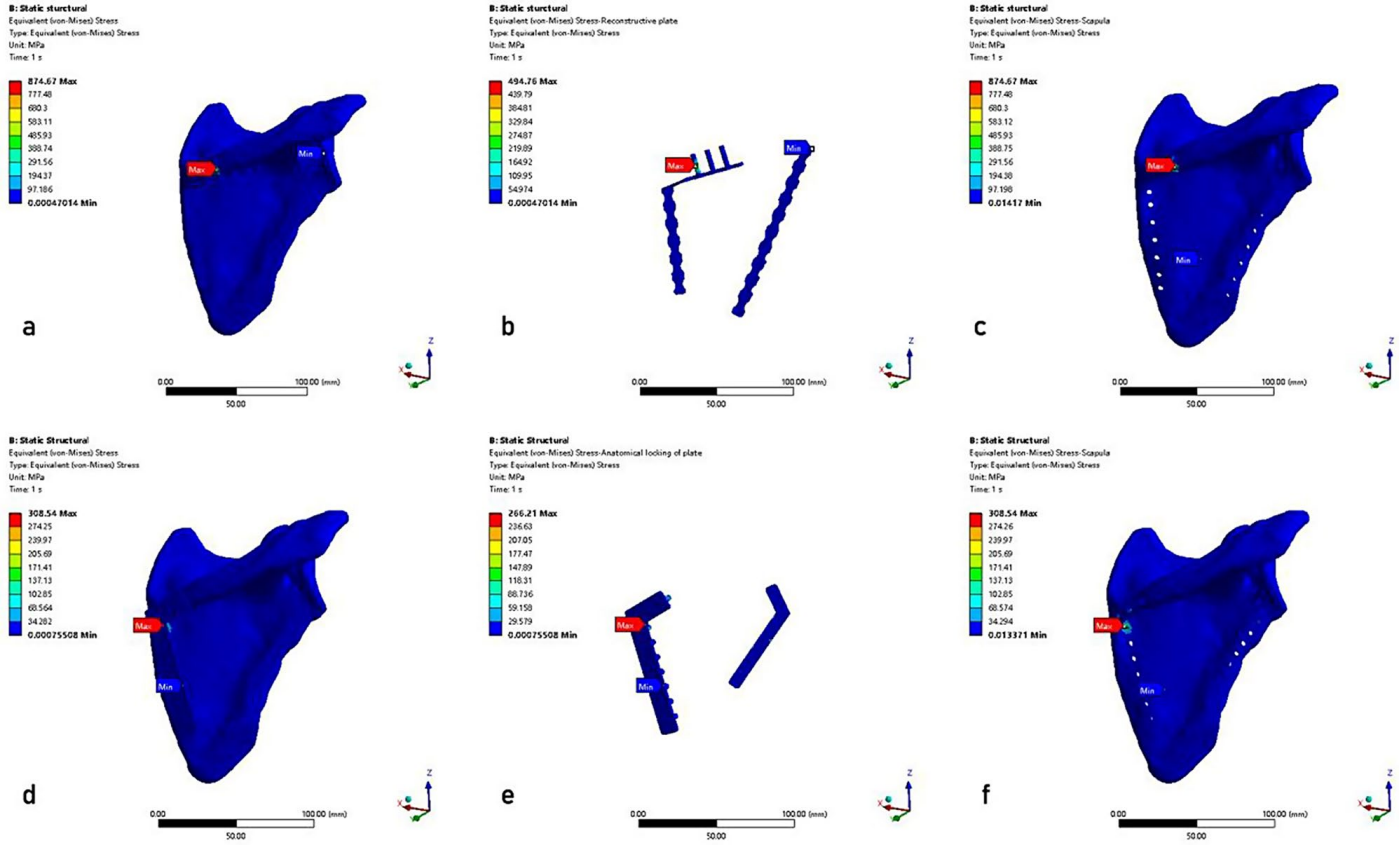
The equivalent von Mises stress distributions. **a**-**c**: RP model (scapula model with reconstructive plates, steel plates, scapula); **d**-**f**: AP model (scapula model with anatomical locking plates, steel plates, scapula)

**Fig. 7:**
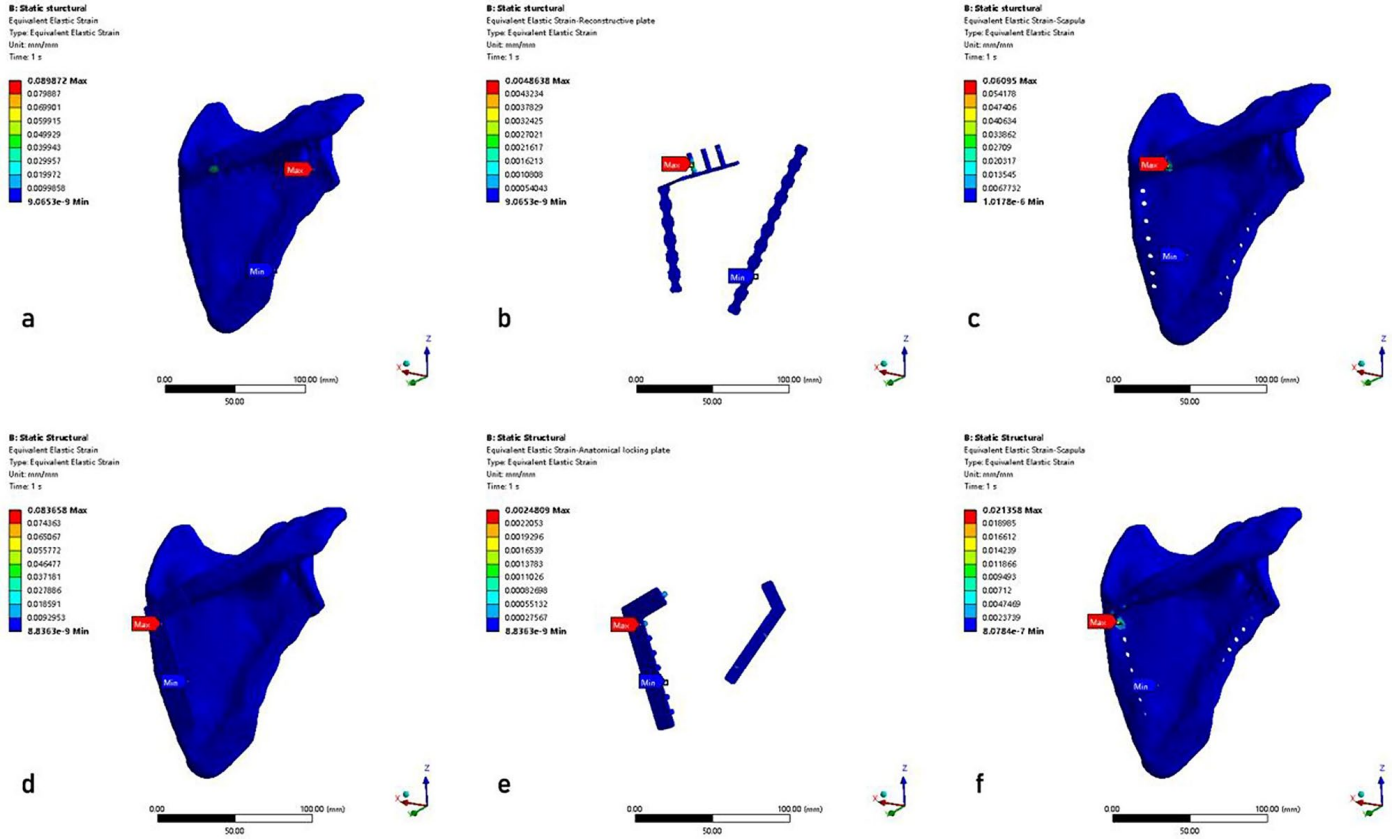
The equivalent elastic strain distributions. **a**-**c**: RP model (scapula model with reconstructive plates, steel plates, scapula); **d**-**f**: AP model (scapula model with anatomical locking plates, steel plates, scapula)

**Fig. 8:**
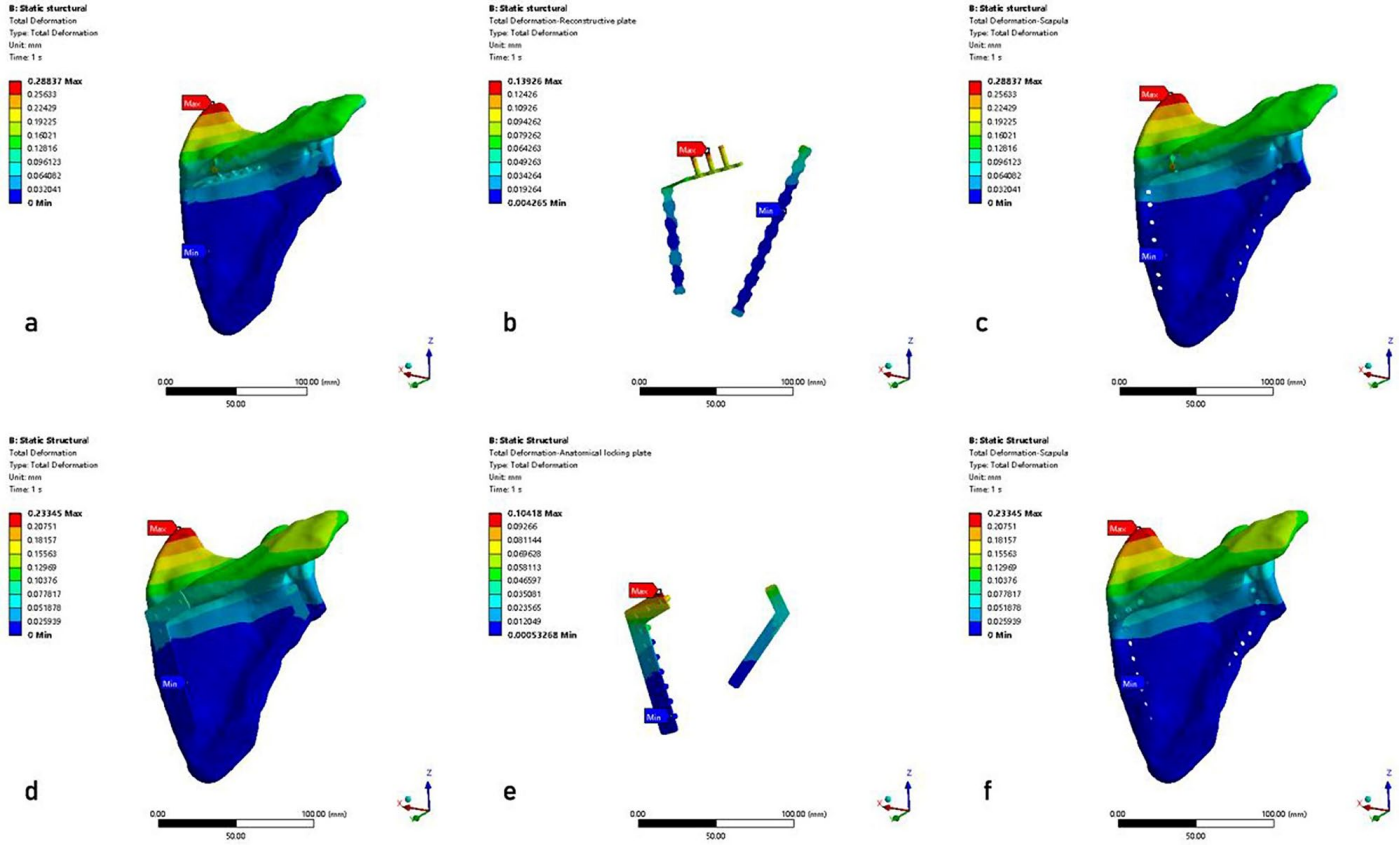
The total deformation of the two models. **a**–**c**: RP model (scapula model with reconstructive plates, steel plates, scapula); **d**–**f**: AP model (scapula model with anatomical locking plates, steel plates, scapula)

The results of the FEA were presented in the equivalent von Mises stress distribution, the equivalent elastic strain distribution, and the total deformation, which were shown in Figs. 6 and 7, and 8, respectively. A tabulated comparison was presented in Table 3, showing the maximum stress, the maximum strain, and the maximum deformation observed in both RP and AP models. Furthermore, the results of the stress, strain, and total deformation were obtained using a consistent color bar range, enabling immediate comparison in terms of rainbow colors that represent the distribution [34].

**Table 3:** Overview of the maximum stress, strain, and deformation values observed in both the RP and AP models

Model	Max. Equivalent (von Mises) stress (MPa)		Max. Equivalent elastic strain (mm/mm)		Max. Deformation (mm)	
	Scapula model with plates	Steel plates	Scapula model with plates	Steel plates	Scapula model with plates	Steel plates
RP	874.67	494.76	0.08987	0.00486	0.28837	0.13926
AP	308.54	266.21	0.08366	0.00248	0.23345	0.10418

### Equivalent von Mises stress and equivalent elastic strain distribution

Figure 6; Table 3 showed the equivalent von Mises stress distributions for the scapula model with plates, steel plates, and scapula. As shown in Fig. 6; Table 3, the maximum von Mises stress value of the AP model was 308.54 MPa, with the highest stress occurring at the edge of the screw hole along the medial plate fracture line. The maximum von Mises stress value of the RP model was 874.67 MPa, and the maximum von Mises stress occurred at the edge of the screw hole in the leading edge of the medial plate. The maximum von Mises stress values of the anatomical plates and reconstructive plates were 266.21 MPa and 494.76 MPa, respectively.

By comparing the results in Fig. 7; Table 3, the maximum equivalent elastic strain values of the RP model and AP model were 0.08987 and 0.08366, respectively. Notably, the AP model exhibited comparatively smaller values compared to the RP model. The anatomical locking plates exhibited a maximum strain that was only half of the reconstructive plates (0.00248 vs. 0.00486). The maximum strain of the anatomical locking plates occurred at the edge of the screw hole near the fracture line (Fig. 7e). The maximum strain of the reconstructive plates occurred at the edge of the third screw hole of the medial plate (Fig. 7b).

### Total deformation

As shown in Fig. 8and Table 3, the maximum displacement of the two models presented similar values (AP model: 0.23345 mm; RP model: 0.28837 mm). Thus, the AP model offered similar stability as the RP model. Meanwhile, the maximum displacement of the scapula body was consistently below 2 mm in each model, thus affirming the reliability of both internal fixation devices [32]. Furthermore, the acromion was found to have the maximum displacement in each group. The primary function of the scapula was to facilitate the movement of the upper limb. The maximum displacement on the models was at the acromion, which was also in line with the anatomical characteristics of the scapula movement.

## Discussion

We described the application of a finite element method to the computation of the scapular body fractures. The method accounted for the contribution of anatomical locking plates for the fixation of scapular body fractures.

FEA has become an indispensable tool in the field of biomechanics for evaluating the mechanical behaviour of orthopedic implants [35]. The current study examined the finite element analysis of scapular body fractures treated by anatomical locking plates and reconstructive plates. Based on the findings, in terms of equivalent von Mises stress, equivalent elastic strain, and total deformation, the AP model exhibited superior safety characteristics, enhanced flexibility, and anticipated stability compared with the RP model. This was evidenced by lower maximum stress, lower maximum strain, and equivalent displacement. Thus, the minimally invasive approach combined with an anatomical locking plate for fixing scapula body fractures could reduce displacement and lower stress limits. Therefore, it can be concluded that anatomical locking plates can provide greater strength and stability during the recovery of the scapular fracture. The safety factor of the two plates was further considered (safety factor = yield strength/maximum stress) [32, 36]. The yield strength of titanium alloy is normally 825 MPa [32]. Based on the calculated results, it was shown that the safety factor of the anatomical locking plate was higher than that of the reconstructive plate. The findings are consistent with those reported by Shang et al. However, the differences between our study and that of Shang lie in the following: (1) the position of the fracture is different. (2) The surgical approach is different. (3) The fixation position of the anatomical plate is different, and the fixation position in this study makes internal fixation easier and reduces soft tissue injury.

Open reduction and internal fixation has been shown good clinical results for patients with traumatic scapular fractures who met the surgical indications [37]. The posterior Judet approach required a large skin incision and disruption of the muscles [38, 39]. The modified Judet approach has demonstrated a high level of achieving optimal reduction for scapular fractures [40]. Nevertheless, it still required an extensive cutaneous. The minimally invasive approach which was used for scapular fractures was a less invasive surgical technique with minimal muscular [18, 41]. The minimally invasive approach minimized the surgical window and the disruption of the posterior scapula musculature. It could be appropriate for patients with scapula body, neck, and posterior glenoid fractures [18]. In this study, a minimally invasive approach was used in the open reduction and internal fixation of scapular body fractures. Furthermore, we modified the application of the plate according to the anatomical structure of the scapula. The angle of the modified medial plate was set at 105 degrees, and the cephalic side of the plate was placed on the scapular spine instead of its lower edge, ensuring a more secure fixation by screwing through the scapular spine. The design could reduce soft tissue injury and was relatively simple to perform during the operation. The modified outer plate was fixed at 120 degrees, which can also take into account for a portion of the lower glenoid fractures. The combination of modified medial and lateral anatomic locking plates with a minimally invasive approach has certain advantages in reducing soft tissue injuries and less intraoperative blood loss.

In this study, we conducted a comprehensive FEA of the anatomical locking steel plates for scapular body fractures. Scapular body fractures are relatively rare but can have significant implications on shoulder function and overall quality of life. The use of locking plates has gained popularity in recent years due to their ability to provide stable fixation and promote early mobilization. By simulating the loading conditions experienced by the scapula, we were able to assess the stress distribution and deformation patterns of the locking plate system. Our results indicated that the anatomical locking plate provided adequate stability and support for scapular body fractures, with minimal risk of implant failure or secondary displacement. This study contributes valuable insights into the biomechanical performance of locking plate systems for scapular body fractures, which can inform clinical decision-making and improve patient outcomes. Further research is needed to validate these findings through clinical trials and long-term follow-up studies.

However, the limitations of this study should also be considered. First, because the FEA is a simulation analysis, the results need to be confirmed by more clinical validation. Second, there were no clinical studies that investigated the effectiveness and rate of the locking plate technique in this study. Third, the FEA may not fully capture the complex nature of bone fractures and healing processes. So, further research is needed and confirmed by clinical trials.

## Conclusion

The study revealed that the minimally invasive approach combined with an anatomical locking plate for scapular body fractures resulted in an improved load distribution and reduced stress concentration at the fracture site. The anatomical locking plate may provide better biomechanical support and stability. These findings underscore the importance of utilizing advanced fixation techniques for optimal outcomes in the management of scapular body fractures. Further research and clinical studies are warranted to validate these results and explore potential applications in orthopedic practice.

## Data Availability

No datasets were generated or analysed during the current study.
